# Bioassembly of Region‐Specific Fibrocartilage Microtissues to Engineer Zonally Defined Meniscal Grafts

**DOI:** 10.1002/adhm.202502208

**Published:** 2025-08-21

**Authors:** Gabriela S. Kronemberger, Kaoutar Chattahy, Francesca D. Spagnuolo, Aliaa S. Karam, Daniel J. Kelly

**Affiliations:** ^1^ Trinity Centre for Biomedical Engineering Trinity Biomedical Sciences Institute Trinity College Dublin Dublin D02 PN40 Ireland; ^2^ Department of Mechanical Manufacturing and Biomedical Engineering School of Engineering Trinity College Dublin Dublin D02 PN40 Ireland; ^3^ Advanced Materials and Bioengineering Research Centre (AMBER) Royal College of Surgeons in Ireland and Trinity College Dublin Dublin D02 PN40 Ireland; ^4^ Department of Anatomy and Regenerative Medicine Royal College of Surgeons in Ireland Dublin D02 YN77 Ireland

**Keywords:** bioassembly, biofabrication, building‐blocks, microtissues, progenitor cell

## Abstract

Meniscal injuries are common orthopaedic problems which can impair knee function and lead to the development of osteoarthritis. While recent advances in tissue engineering have enabled the fabrication of meniscus‐like grafts, these do not fully replicate the zonal structure and composition of the native meniscus. Here, fibrocartilage microtissues are used as building blocks to biofabricate zonally defined meniscal grafts. Meniscus progenitor cells (MPCs) isolated from the inner (iMPC) and outer (oMPC) regions of caprine menisci are used to engineer region‐specific meniscal microtissues. Both iMPC and oMPC‐derived microtissues are rich in glycosaminoglycans (GAGs) and collagen, with iMPC‐derived microtissues staining more intensely for type II collagen. These microtissues are assembled into two different physically confining moulds, where they rapidly fuse and generate a fibrocartilaginous graft. The impact of the catabolic enzyme chondroitinase‐ABC (cABC) on the composition and structural organization of the meniscal grafts is then explored, which is found to reduce sGAG levels but to increase collagen fiber thickness. Finally, iMPC and oMPC microtissues are spatially assembled to biofabricate a scaled‐up and zonally defined meniscal construct. These findings support the use of MPC‐derived microtissues as biological building blocks for the engineering of meniscal grafts.

## Graphical abstract

Meniscal microtissues are fabricated using progenitor cells isolated from the inner (iMPCs) and outer (oMPCs) regions of the meniscus. Meniscal microtissues fuse into cohesive constructs rich in sGAG and collagen. cABC treatment modulates collagen fiber formation and organization in assembled grafts. Zonally defined meniscal grafts are engineered via the spatial assembly of iMPC and oMPC‐derived microtissues.

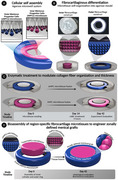

## 1 Introduction

The meniscus is a crescent‐shaped fibrocartilage structure which plays a crucial role in load transmission and stability within the knee joint.^[^ ^1^ ^]^ Meniscal injuries are amongst the most frequent knee pathologies and can significantly increase the risk of developing osteoarthritis.^[^ ^2^, ^3^ ^]^ Current clinical treatments, such as meniscectomy, provide short term symptomatic relief but dramatically reduce meniscal functionality and accelerate the progression of osteoarthritis.^[^ ^4^ ^]^ Alternative approaches, including meniscal allograft transplantation, synthetic meniscal substitutes such as Actifit, and autologous chondrocyte implantation (ACI), have all been reported as treatment options for meniscal injuries,^[^ ^4^, ^5^, ^6^ ^]^ however, long‐term outcomes remain poor. These challenges have motivated increased interest in the field of meniscus tissue engineering.^[^ ^7^ ^]^

The meniscus consists of two anatomically and functionally distinct regions: the inner and outer zones.^[^ ^8^ ^]^ The extracellular matrix (ECM) of the outer region is rich in type I collagen, which is primarily organized in a circumferential manner, allowing the tissue to withstand large tensile forces during joint movement. The tensile modulus of the outer region of the meniscus can exceed 100 MPa in the circumferential direction.^[^ ^9^ ^]^ In contrast, the inner zone contains higher levels of type II collagen and sulphated glycosaminoglycans (GAGs), making it more suited to resisting compressive loads. The compressive modulus of the inner zone is comparable to that of articular cartilage, typically ranging from 100 kPa to 1 MPa.^[^ ^10^ ^]^ The meniscus is partially vascularized, with blood vessels largely restricted to the periphery (10–30%) of the outer region, facilitating greater intrinsic healing capacity, while the inner zone remains avascular and largely aneural, making it less capable of spontaneous repair.^[^ ^11^, ^12^ ^]^ Accurately replicating this zonal organization in engineered grafts is critical to successful regeneration, as constructs that emulate the native composition and architecture are better positioned to restore the intricate load‐bearing and stabilizing functions of the meniscus, ultimately enhancing their potential for functional joint restoration and long‐term success.^[^ ^13^ ^]^ While the use of emerging technologies such as controlled growth factor delivery,^[^ ^14^ ^]^ enzymatic treatments,^[^ ^15^, ^16^ ^]^ novel biomaterials,^[^ ^14^ ^]^ 3D bioprinting^[^ ^17^, ^18^, ^19^ ^]^ and bioreactors for biophysical stimulation^[^ ^20^, ^21^ ^]^ have enabled the engineering of more complex meniscal constructs, these strategies typically fail to generate grafts that recapitulate the zonal structure and composition of the native tissue.

Bottom‐up tissue engineering strategies aim to create complex constructs that better mimic the structure and function of native tissues, often using multiple self‐organized cellular units such as cellular aggregates, microtissues, or organoids as biological building‐blocks to generate scaled‐up grafts.^[^ ^22^, ^23^ ^]^ Numerous studies have explored the potential of such microtissues to engineer musculoskeletal tissues such as bone^[^ ^24^, ^25^, ^26^, ^27^ ^]^ and cartilage.^[^ ^28^, ^29^, ^30^, ^31^, ^32^ ^]^ Such approaches are particularly attractive for the engineering of spatially heterogeneous tissues like the meniscus, where phenotypically distinct microtissues can potentially be engineered separately and then assembled to generate larger, spatially complex tissues or organs. Realising this goal first requires the identification of a cell type capable of generating microtissues mimetic of the different regions of the native meniscus. While mesenchymal stem/stromal cells (MSCs) are commonly used in the development of cartilage and bone forming microtissues, it remains unclear whether this cell source is capable of generating meniscus‐specific ECM. Meniscus progenitor cells (MPCs), which can be isolated from the inner and outer regions of the meniscus,^[^ ^33^, ^34^, ^35^ ^]^ offer a distinct advantage for producing meniscal microtissues due to their inherent ability to maintain a meniscal phenotype during monolayer expansion and subsequently produce zone‐specific meniscal tissue.^[^ ^36^, ^37^, ^38^ ^]^ Another key challenge in the field of meniscus tissue engineering is the generation of grafts with a collagen content and organization similar to the native tissue. This can potentially be addressed using the temporal application of extracellular matrix (re)modeling enzymes such as chondroitinase ABC (cABC) and lysyl‐oxidase (LOX) in culture,^[^ ^15^, ^39^, ^40^ ^]^ which have been shown to modulate tissue composition and improve collagen organisation.^[^ ^41^, ^42^, ^43^ ^]^ Furthermore, we have previously shown that cABC treatment enhanced the (re)modeling of hyaline cartilage grafts engineered via the fusion of articular cartilage microtissues, leading to the generation of more hierarchically organized structures.^[^ ^44^ ^]^ Such enzymatic treatments could also potentially be used to improve the functionality of meniscal grafts engineered via the fusion of multiple fibrocartilage microtissues.

Traditional meniscus tissue engineering strategies typically support the development of homogeneous constructs that fail to accurately replicate the zonal composition and organization of the native tissue. Such challenges could potentially be addressed using bottom‐up tissue engineering strategies that aim to replicate key aspects of normal developmental processes to generate more biomimetic grafts. In the context of meniscus tissue engineering, this could potentially be achieved by providing phenotypically distinct meniscal microtissues with the appropriate environmental cues to fuse and (re)model into heterogeneous grafts that more closely resemble the native meniscus. Therefore, the goal of this study was to biofabricate zonally defined meniscus grafts using zone‐specific fibrocartilage microtissues generated using MPCs. To this end, MPCs from the inner (iMPC) and outer (oMPC) regions of the menisci were first isolated to engineer region‐specific meniscal microtissues in a medium‐high throughput manner. iMPC and oMPC‐derived microtissues were then assembled in different spatial configurations to assess their capacity to fuse and develop region‐specific meniscal tissue. We then explored the impact of cABC treatment on the composition, structural organization, and overall collagen levels of the meniscal grafts. Finally, iMPC and oMPC microtissues were combined and allowed to fuse into a single construct, with the goal of engineering a cohesive, yet zonally defined, meniscus graft.

## 2 Results

### 2.1 Isolation of Meniscal Progenitor Cells from the Inner and Outer Region of the Meniscus

**Figure 1:**
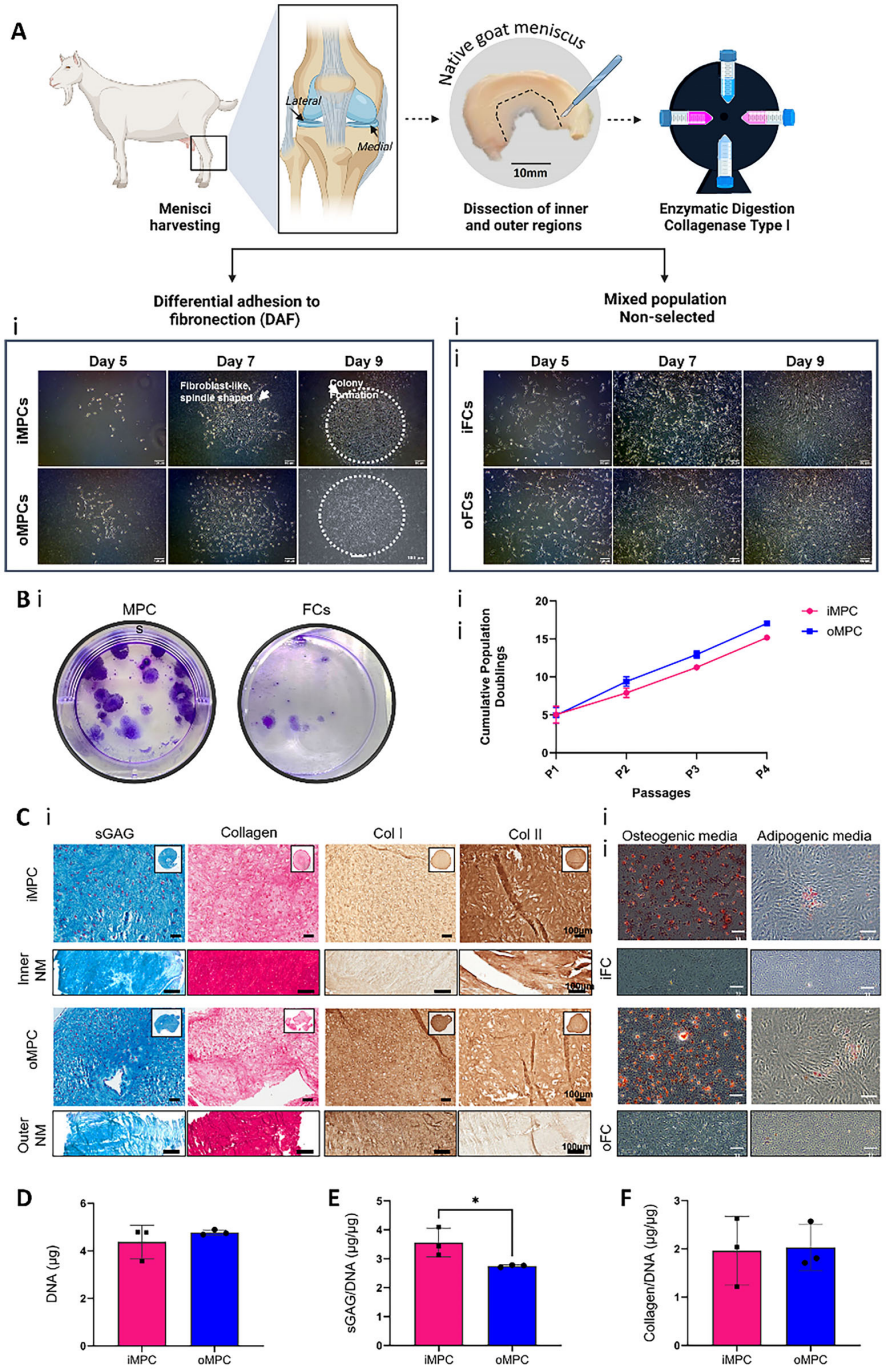
Isolation, morphological comparison, and tri‐lineage differentiation potential of inner and outer meniscus progenitor cells (iMPCs and oMPCs). A) Schematic illustrating the isolation of meniscus progenitor cells (MPCs) and fibrochondrocytes (FCs). Morphological comparison of MPCs (Ai) and FCs (Aii) isolated from goat meniscal tissue at passage 0 (P0) on days 5, 7, and 9 in monolayer culture (scale bar = 100 µm). B) Crystal violet staining to visualize colonies in MPCs and FCs (i). Cumulative population doublings of iMPCs and oMPCs across passages 1, 2, 3, and 4 (ii). Ci) Histological and immunohistochemical staining of iMPC and oMPC pellets at passage 3: sulphated glycosaminoglycan (Alcian Blue, AB) and total collagens (Picrosirius Red, PR), collagen types I and II after 21 days of chondrogenic culture, with native meniscus (NM) controls (scale bar = 100 µm). Cii) Alizarin red staining for mineralization and Oil Red O staining for lipid deposits in iMPC, oMPC, iFC, and oFCs following 3 weeks of osteogenic and adipogenic differentiation (scale bar = 100 or 200 µm). D) DNA, E) sGAG, and F) collagen content after 21 days of chondrogenic culture. N = 3, mean ± SD. The asterisks indicate p‐values obtained by a nonpaired student *t*‐test (^*^ *p* <0.05).

Caprine meniscus progenitor cells (MPCs) were isolated based on their ability to adhere to fibronectin‐coated plates within 20 min after seeding. Both inner and outer MPCs began to spread by day 5, forming colonies by day 7 that continued to expand with time in culture (**Figure** **1**A‐i). No obvious differences in cellular morphology were observed between inner and outer progenitors or the mixed population of fibrochondrocytes (FCs). However, MPCs exhibited a greater colony‐forming capacity compared to FCs (Figure 1A‐ii), with MPCs observed to produce larger diameter colonies compared to FCs (Figure 1B‐i). Cumulative population doublings demonstrate the strong proliferative capacity of iMPCs and oMPCs, with a linear increase in cumulative population doublings from passage 1 to passage 4 (Figure 1B‐ii). Both inner and outer MPCs could generate a calcified matrix following 21 days in osteogenic medium, as indicated by positive alizarin red staining. Lipid droplet formation was also observed in MPCs following 21 days in adipogenic media, though with only a few vacuoles. In contrast, iFCs and oFCs did not show a potential to undergo osteogenic or adipogenic differentiation, as evidenced by the absence of mineral deposition and lipid formation under the relevant culture conditions (Figure 1C‐ii). The fibrochondrogenic capacity of iMPC and oMPC was assessed in pellet culture using passage 3 cells maintained in chondrogenic medium. Both MPC populations exhibited a fibrochondrogenic phenotype, as demonstrated by the histological analysis where strong staining for sGAG and collagen deposition was observed (Figure 1C‐i). Notably, MPCs at a higher passage (passage 4) maintained their fibrochondrogenic potential, as evidenced by the strong staining for collagen and sGAGs (data not shown). Immunohistochemical analysis revealed differences between iMPCs and oMPCs in their fibrocartilage phenotype (Figure 1C‐i). Tissues generated by iMPCs stained more intensely for collagen type II, while tissues produced by oMPCs were positive for both collagen types I and II. This staining pattern closely mirrors that observed in the collagen staining of native meniscus controls for each respective region. No significant difference in DNA content was observed between iMPC and oMPC‐derived pellets (Figure 1D). iMPCs secreted significantly higher levels of sGAG compared to oMPCs, again mimicking the zonal differences observed in the native meniscus tissue (*p* <0.05) (Figure 1E). No statistical differences in total collagen content were observed in tissues generated by iMPC and oMPC (Figure 1F).

### 2.2 Biofabrication of Zonally Defined MPC Derived Microtissues

**Figure 2:**
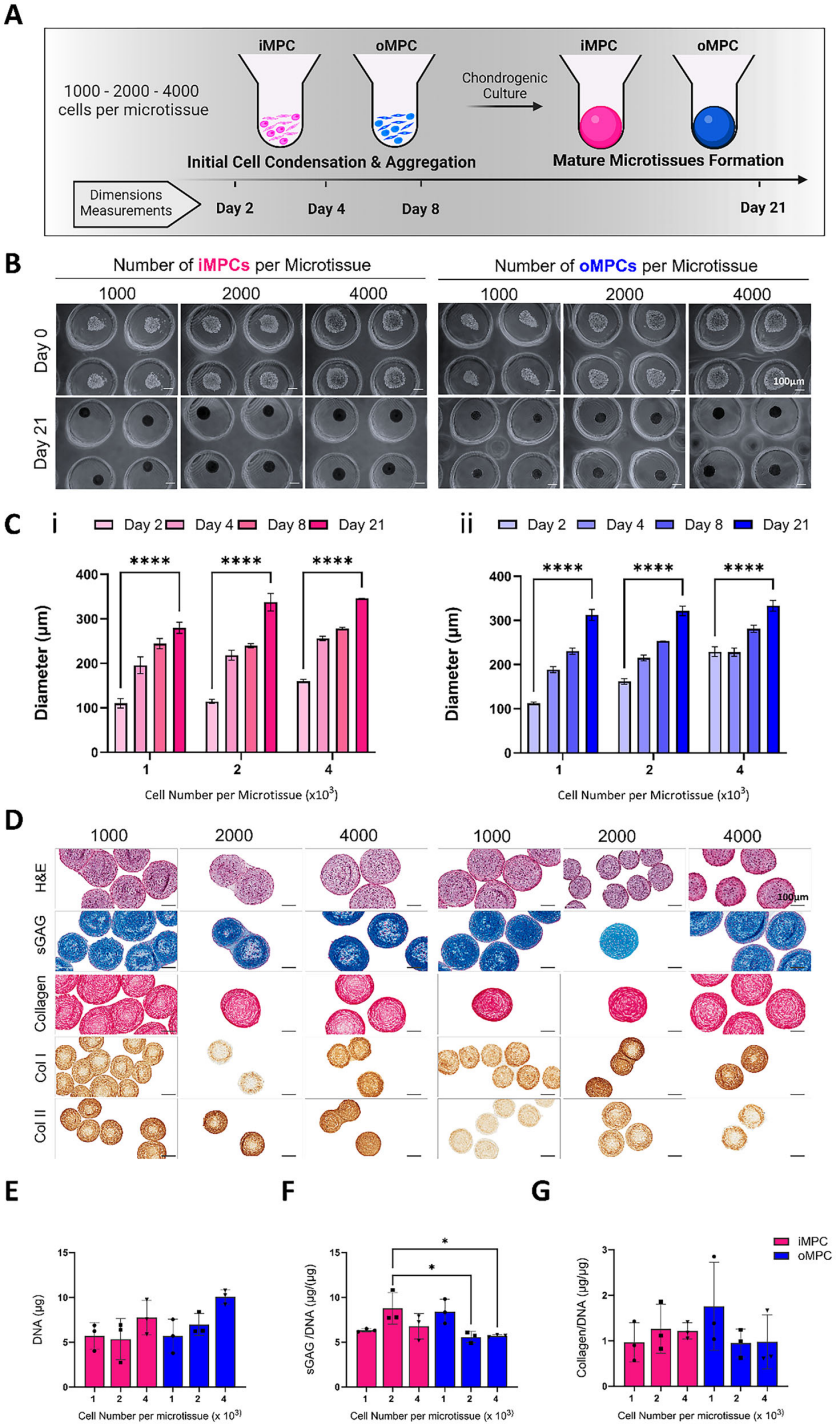
Biofabrication and characterisation of iMPC and oMPC derived microtissues. A) Schematic illustrating the initial cell seeding densities of iMPCs and oMPCs in individual microwells for condensation and subsequent microtissue formation using a 401‐microwell mould. B) Brightfield microscopy images of biofabricated iMPC‐ and oMPC‐derived microtissues at days 0 and 21 of chondrogenic culture, shown at three different cell densities (1000, 2000, and 4000 cells microtissue^−1^) (scale bar = 100 µm). C) Quantification of iMPC‐ (i) and oMPC‐ (ii) derived microtissue diameters over the chondrogenic culture period. Error bars denote standard deviation. D) Histological and immunohistochemical analysis of iMPC‐ and oMPC‐derived microtissues after 21 days of chondrogenic induction (scale bar = 100 µm). E–G) Biochemical quantification of DNA (E), sGAG (F), and collagen (G) content after 21 days of chondrogenic differentiation. N = 3, mean ± SD. The asterisks indicate p‐values obtained by nonpaired two‐way ANOVA followed by Tukey's multiple comparisons post‐test (^****^ *p* <0.001) and by nonpaired one‐way ANOVA followed by Kruskal–Wallis multiple comparisons post‐test (^*^ *p* <0.05).

Region‐specific meniscal microtissues could potentially be used as biological building blocks for the biofabrication of zonally defined meniscal grafts. To this end, we attempted to generate meniscal microtissues using a range of different starting MPC numbers (1000, 2000, and 4000) per microtissue. On day 0, cells were evenly distributed throughout the microwells, resembling a cell suspension (**Figure** **2**B). By day 2, small cell aggregates were observed in all groups, with microtissue size increasing in proportion to the initial cell number. All groups continued to grow throughout the culture period, with larger aggregates particularly evident for the higher seeding density (4000 cells per microwell) (Figure 2B). After 21 days of culture, microtissues derived from both iMPCs and oMPCs displayed a uniform and homogeneous morphology at all cell densities, with their size increasing as the initial cell number rose (Figure 2C). Histological staining revealed consistent matrix deposition across groups, with collagen fibers prominently aligned along the edges of the microtissues in a circular pattern. Type II collagen was more pronounced in the iMPC‐derived microtissues, while type I collagen staining was more intense in oMPC‐derived microtissues, confirming the development of meniscal microtissues with region‐specific characteristics (Figure 2D). sGAG synthesis was significantly higher (*p* <0.05) in the 2000‐cell iMPC‐derived microtissues compared to the 2000 and 4000‐cell oMPC‐derived microtissues (Figure 2F). Total collagen synthesis was not significantly affected by the initial number of MPCs per microtissue or the MPC source (Figure 2G).

### 2.3 The Fusion of iMPC and oMPC Derived Microtissues Produces Phenotypically Distinct Engineered Constructs

**Figure 3:**
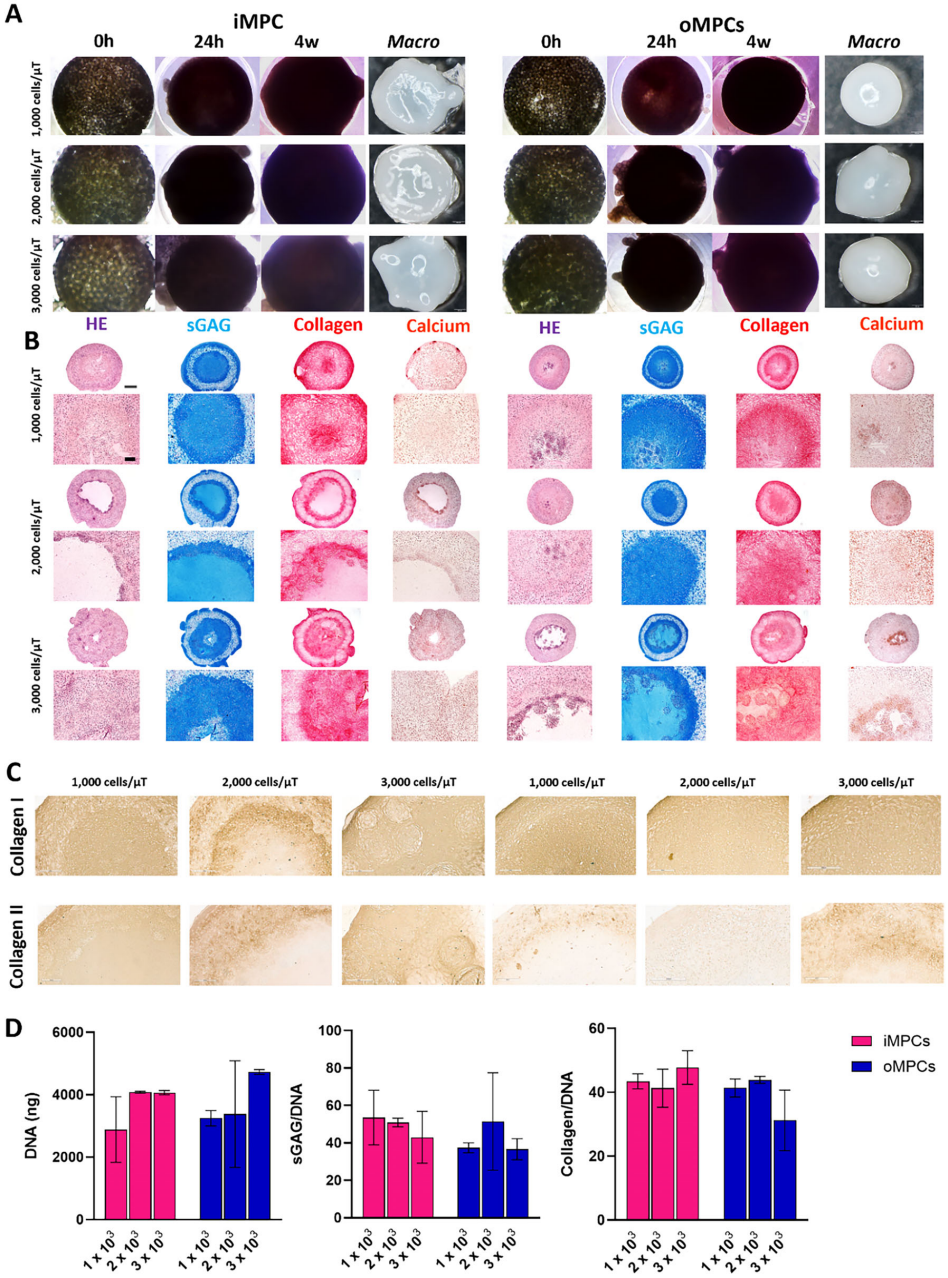
iMPC and oMPC assembled microtissues have differences in phenotype when assembled in the cylindrical shape mould. A) Phase contrast images of iMPC and oMPC microtissues fabricated using densities of 1 × 10^3^, 2 × 10^3,^ and 3 × 10^3^ cells at 0 h, 24 h, and 4 weeks. Macroscopic images of constructs at 4 weeks of culture. B) Hematoxylin and Eosin (HE), Alcian Blue (sGAG), Picrosirius Red (Collagen), and Alizarin Red (Calcium) stains of iMPC and oMPC assembled microtissues at 4 weeks of culture. (B) Immunohistochemistry of collagens I and II of assembled microtissues at 4 weeks of culture. C) Biochemical quantification of total DNA, sGAG/DNA, and collagen/DNA of iMPC and oMPC assembled microtissues at 4 weeks of culture. N = 3, mean ± SD. Scale bars: (A) −50 µm; (B) 1 mm and 100 µm; (C) −200 µm.

The fusion of iMPC and oMPC microtissues in a cylindrical mould was next investigated over a period of 4 weeks in media supplemented with TGF‐β3 (**Figure** **3**). iMPC and oMPC‐derived microtissues were fabricated at densities of 1000, 2000, and 3000 cells µT^−1^. Fusion of both iMPC and oMPC‐derived microtissues occurred within 24 h (Figure 3A). Macroscopic images of iMPC and oMPC microtissue‐derived constructs after 4 weeks of culture show the formation of a compact tissue structure, closely resembling the intended shape defined by the outer mould (Figure 3A).

The potential of the fused iMPC and oMPC microtissue constructs to form scaled‐up fibrocartilage tissue was assessed using histological, immunohistochemical, and biochemical analyses (Figure 3B–D). Following 4 weeks of in vitro culture, robust fibrochondrogenesis was observed in all iMPC and oMPC‐derived constructs, as evident by alcian blue staining for sGAG deposition and picrosirius staining for collagen deposition (Figure 3B). Small nodes of calcification were occasionally observed in iMPC and oMPC‐derived constructs (Figure 3B). Collagen deposition was typically more pronounced in the outer region of the iMPC and oMPC constructs (Figure 3B). The constructs generated using iMPC‐derived microtissues stained more intensely for collagen type II (Figure 3C). The constructs generated using oMPC microtissues generally stained more homogenously for type I collagen (Figure 3C). Total sGAG and collagen synthesis was comparable in iMPC and oMPC microtissue‐derived constructs (Figure 3D).

### 2.4 Generation of Ring‐Shaped Meniscal Grafts using MPC Derived Microtissues

**Figure 4:**
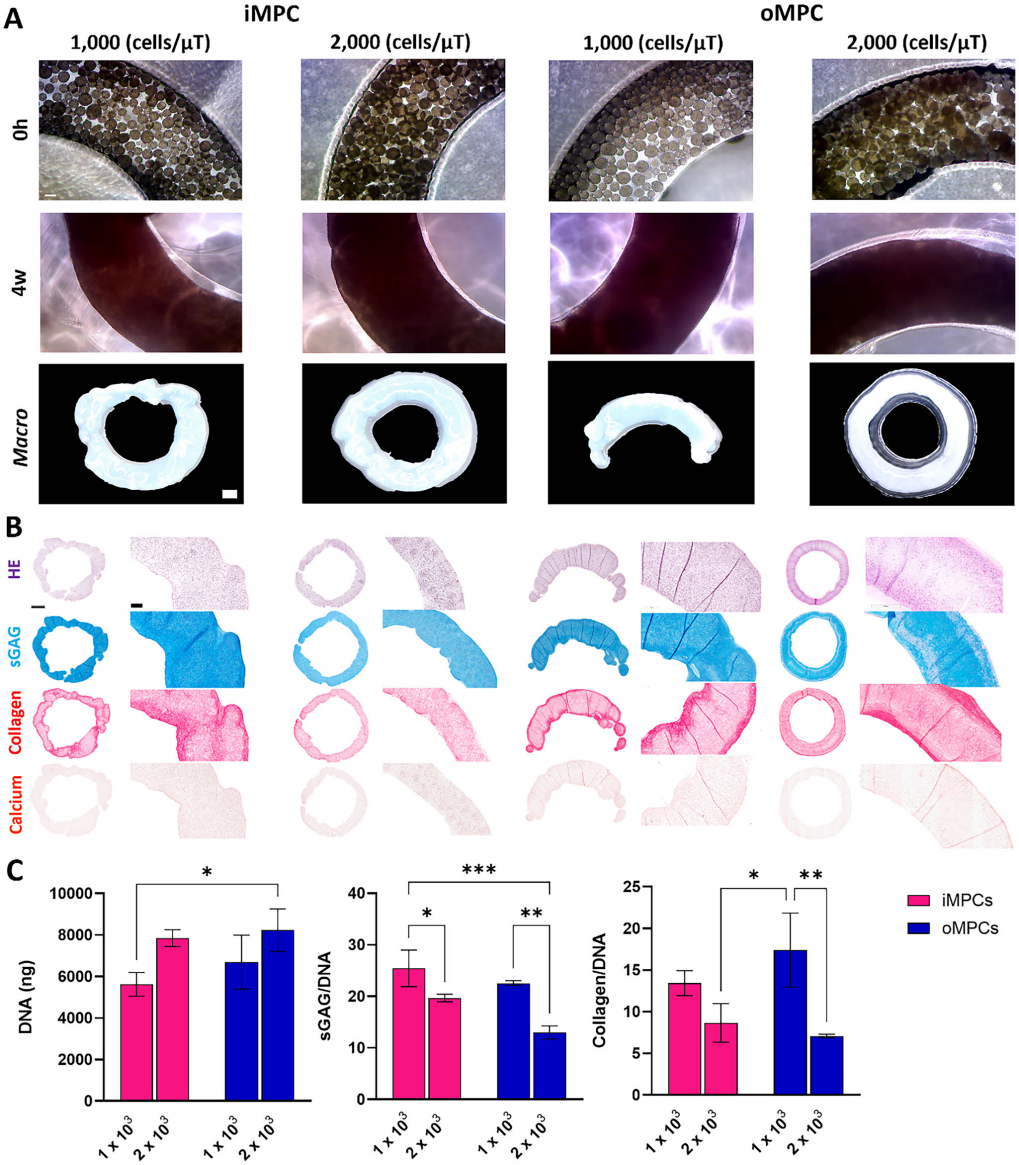
iMPC and oMPC microtissues self‐organized in a ring shape and maintain differences in phenotype. A) Phase contrast images of iMPC and oMPC ring assembled microtissues fabricated using densities of 1 × 10^3^ and 2 × 10^3^ cells at 0 h and 4 weeks. Macroscopic images of iMPC and oMPC ring assembled microtissues at 4 weeks of culture. B) Hematoxylin and Eosin (HE), Alcian Blue (sGAG), Picrosirius Red (Collagen), and Alizarin Red (Calcium) stains of iMPC and oMPC ring assembled microtissues at 4 weeks of culture. C) Biochemical quantification of total DNA, sGAG/DNA, and collagen/DNA of iMPC and oMPC ring assembled microtissues at 4 weeks of culture. N = 3, mean ± SD. The asterisks indicate p‐values obtained by nonpaired two‐way ANOVA followed by Tukey's multiple comparisons post‐test (^*^ *p* <0.05; ^**^ *p* <0.01; ^***^ *p* <0.005). Scale bars: (A) −100 and 500 µm (macro); (B) 1 mm and 200 µm.

Generating a large viable tissue with shape fidelity is a key challenge in meniscus tissue engineering. To address this, we seeded iMPC and oMPC‐derived microtissues into ring‐shaped agarose moulds (**Figure** **4**). As a control, we also seeded bone marrow MSC‐derived microtissues within the same ring mould (Figure S4, Supporting Information). iMPC and oMPC microtissues were fabricated at densities of 1000 and 2000 cells µT^−1^. After 4 weeks of in vitro culture, both iMPC and oMPC‐derived microtissues fused into a dense ring‐shaped tissue (Figure 4A), except for the 1000 oMPCs per microtissue group which contracted in culture. Histological analysis revealed strong sGAG staining and no evidence of calcium deposition in both iMPC and oMPC ring constructs (Figure 4B). When normalized to DNA content, sGAG and collagen deposition were significantly higher in constructs generated using 1000 cells µT^−1^ compared to those formed with 2000 cells µT^−1^ (Figure 4C). While both iMPC and oMPC‐derived constructs exhibited comparable levels of total sGAG and collagen, their matrix composition and structure differed. Specifically, iMPC constructs stained more intensely for type II collagen, whereas oMPC constructs were richer in type I collagen.

In contrast, microtissues derived from MSCs initially fused but contracted into a sphere‐like shape after 4 weeks of culture at both seeding densities (1000 and 2000 cells µT^−1^; Figure S4, Supporting Information). These MSC‐derived aggregates underwent robust chondrogenesis, as evidenced by strong sGAG and collagen staining. It is also important to note that when lower numbers of iMPC or oMPC microtissues were seeded into the moulds, no ring‐shaped constructs were formed (Figure S3, Supporting Information).

### 2.5 Enzymatic Treatment Modulates Collagen Fiber Organization and Thickness

**Figure 5:**
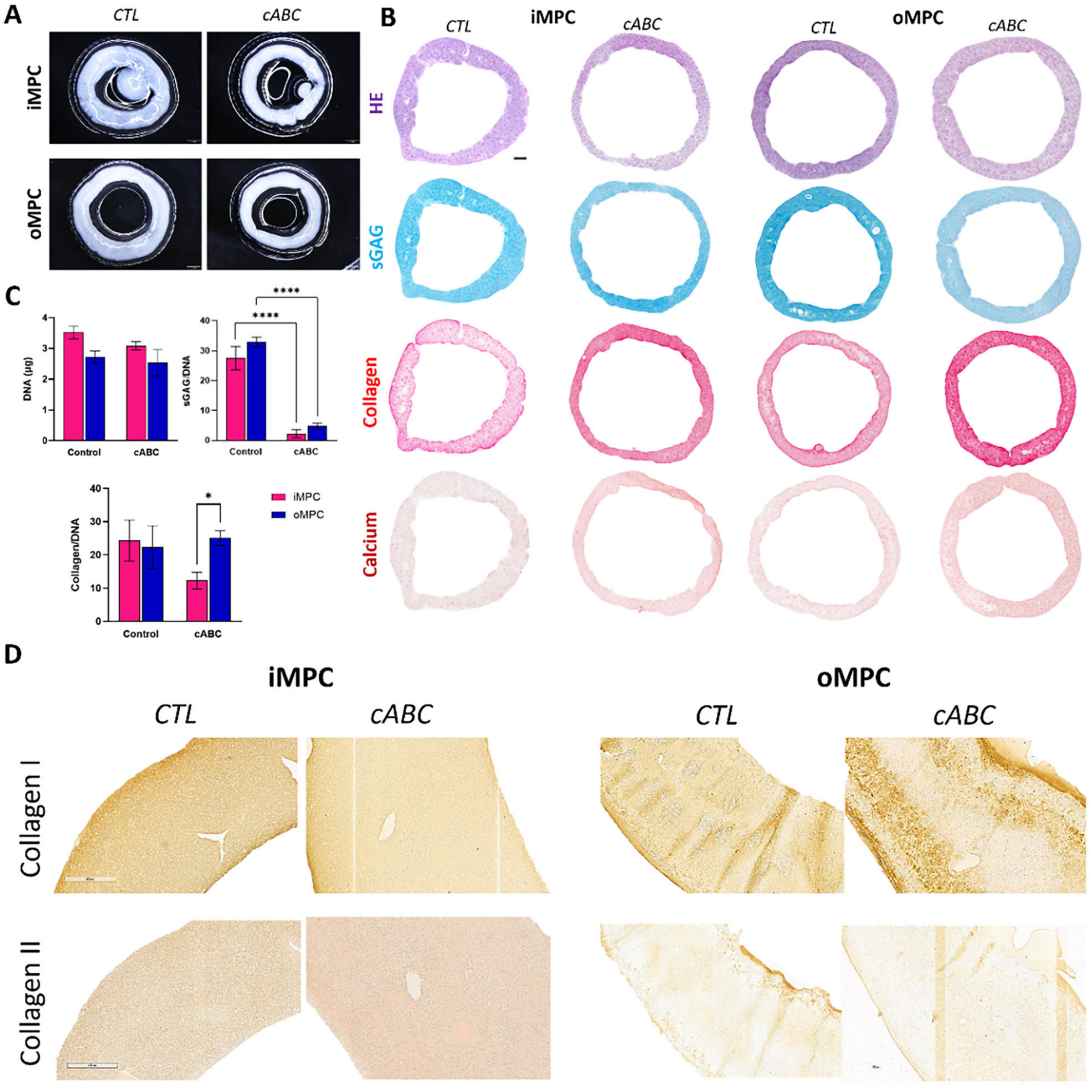
cABC treatment does not impact the phenotype of iMPC and oMPC microtissue‐derived constructs. A) Macroscopic images of untreated and enzymatically treated iMPC and oMPC ring assembled microtissues at 4 weeks of culture. B) Hematoxylin and Eosin (HE), Alcian Blue (sGAG), Picrosirius Red (Collagen), and Alizarin Red (Calcium) stains of untreated and enzymatically treated iMPC and oMPC ring assembled microtissues at 4 weeks. C) Biochemical quantification of total DNA, sGAG/DNA, and collagen/DNA of untreated and enzymatically treated iMPC and oMPC ring assembled microtissues at 4 weeks. D) Immunohistochemistry of collagens I and II of untreated and enzymatically treated iMPC and oMPC assembled microtissues at 4 weeks of culture. N = 3, mean ± SD. The asterisks indicate p‐values obtained by nonpaired two‐way ANOVA followed by Tukey's multiple comparisons post‐test (^*^ *p* <0.05; ^**^ *p* <0.01). Scale bars: (A) −1 mm; (B) −1 mm, and (D) −400 µm.

Since the use of enzymatic agents has shown great promise in enhancing the quality of engineered cartilage^[^ ^42^, ^44^, ^75^, ^76^ ^]^ and meniscus^[^ ^16^, ^18^ ^]^ tissues through modulation of the developing matrix, we next sought to investigate the effect of temporal enzymatic treatment on the functional development of fibrocartilaginous constructs engineered using MPC‐derived microtissues. To this end, we exposed the tissue rings engineered using MPC‐derived microtissues to chondroitinase ABC (cABC) for 4 h on day 14 of chondrogenic culture (**Figure** **5**). Enzymatic treatment did not impact the capacity of MPC‐derived microtissues to generate ring‐shaped tissues (Figure 5A). As expected, after 4 weeks of culture, cABC treatment significantly reduced total sGAG levels compared to untreated controls (Figure 5C). However, no significant differences were observed in DNA or total collagen content following enzymatic treatment (Figure 5C). Histological staining showed that oMPC‐derived constructs, both untreated and cABC‐treated, stained positively for type I collagen but did not exhibit type II collagen staining. In contrast, iMPC‐derived constructs stained positively for both type I and type II collagen, regardless of treatment, indicating a more chondrogenic extracellular matrix profile (Figure 5D). This suggests that cABC treatment does not affect the capacity of iMPC and oMPCs to generate region‐specific meniscal microtissues. Therefore, while cABC treatment influences the composition of the engineered tissues, cell‐specific differences in the deposition of specific collagen sub‐types would appear to persist.

**Figure 6:**
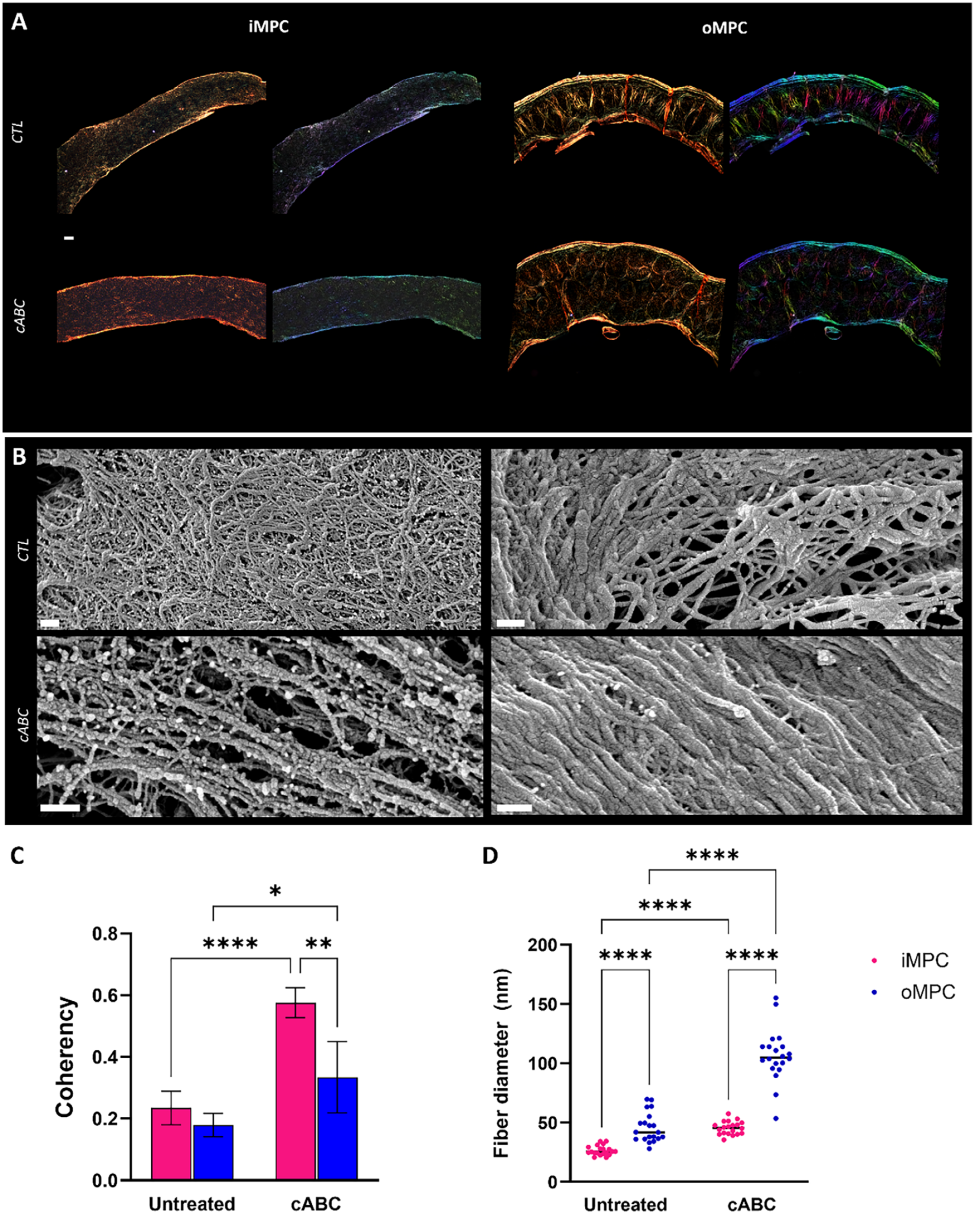
cABC treatment in iMPC and oMPC ring assembled microtissues alters collagen network directionality and increases the thickness of the collagen fibers. A) Polarized light microscopy (PLM) images of untreated and enzymatically treated iMPC and oMPC ring assembled microtissues at 6 weeks of culture. B) Scanning electron microscopy (SEM) images of untreated and enzymatically treated iMPC and oMPC ring assembled microtissues at 4 weeks of culture. C) Collagen fibers coherency quantification, where a value of 1 indicates fibers are aligned in the same direction, while a value of 0 indicates dispersion of fibers in all directions. D) Quantification of the collagen fiber diameter. N = 3, mean ± SD. The asterisks indicate *p*‐values obtained by nonpaired two‐way ANOVA followed by Tukey's multiple comparisons post‐test (^*^ *p* <0.05; ^**^ *p* <0.01). Scale bars: (A) −100 µm; (B) −200 and 300 nm (oMPC under cABC treatment).

Polarized‐light microscopy (PLM) was next used to investigate if enzymatic treatment influences collagen organization within the engineered tissue (**Figure** **6**A). Under polarized light microscopy, the color intensity of collagen fibers showed no noticeable difference between the untreated and cABC‐treated groups (Figure 6A). However, the coherency values (where a value of 1 indicates fibers are aligned in the same direction, while a value of 0 indicates dispersion of fibers in all directions) were significantly higher for the inner (*p* <0.0001) and outer (*p* <0.05) assembled microtissues rings treated with the cABC, indicating that the collagen fibers were more aligned (Figure 6C). To further evaluate the influence of cABC treatment on collagen network development and maturation, the iMPC and oMPC assembled microtissues constructs were analysed by SEM (Figure 6B). Enzymatic treatment appeared to enhance collagen alignment compared to untreated controls (Figure 6B). Furthermore, the collagen fiber diameter (Figure 6D) increased from an average of ≈20 nm in untreated samples to an average of ≈100 nm in enzymatically treated groups (*p* <0.0001). Collagen fiber diameter was significantly higher in oMPC‐derived tissues compared to the iMPC‐derived tissues (*p* <0.0001).

### 2.6 The Spatial Assembly of iMPC and oMPC Derived Microtissues Results in a Zonally Defined Meniscal Graft with Spatially Distinct Phenotypes

**Figure 7:**
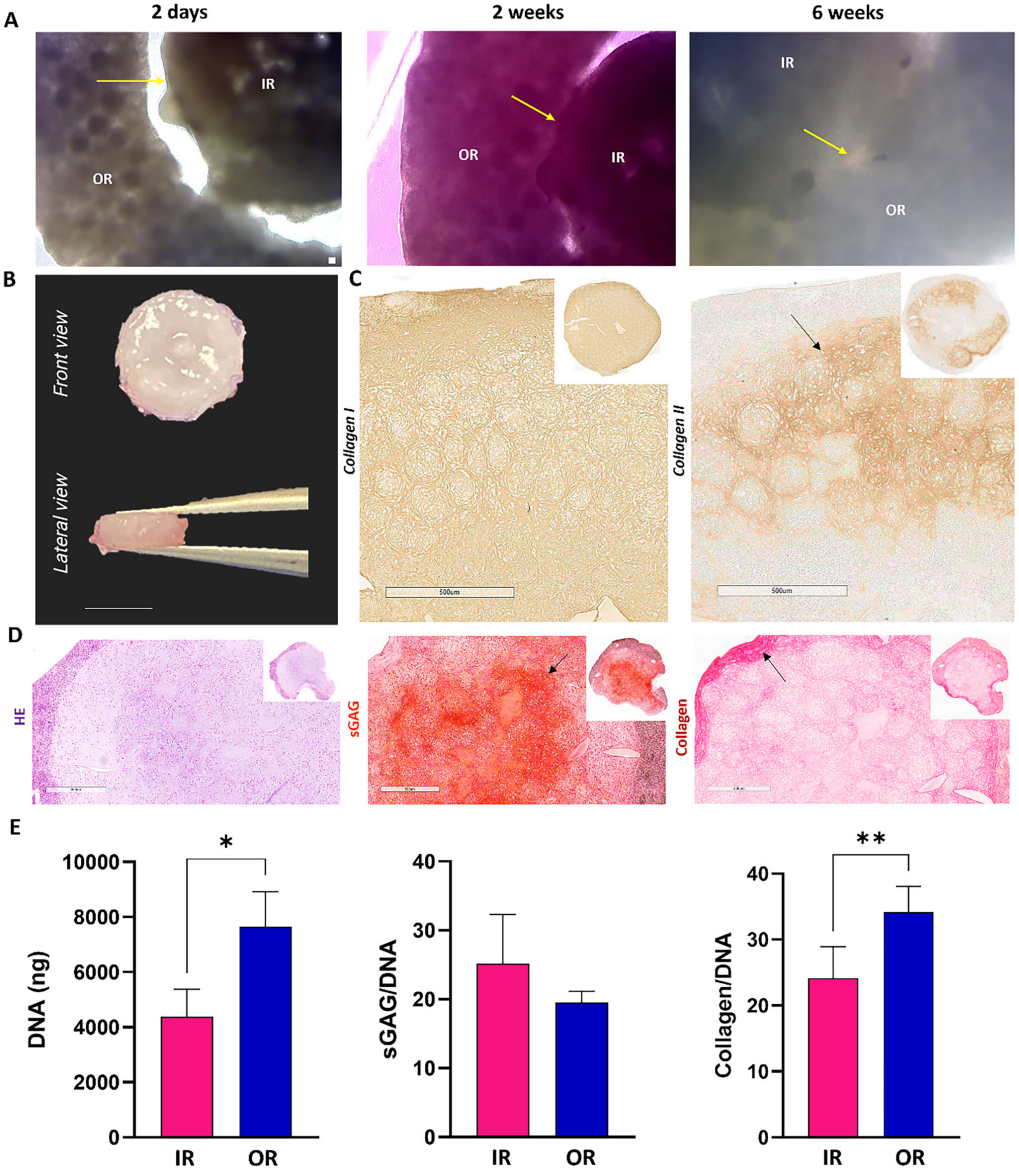
Biofabrication of a zonally defined menisci graft by self‐organization of iMPC and oMPC assembled microtissues. A) Phase contrast images of fused iMPC and oMPC assembled microtissues at 2 days, 2 and 6 weeks. B) Front and lateral macroscopic images of the zonally defined menisci graft. C) Immunohistochemistry of collagens I and II of fused iMPC and oMPC assembled microtissues at 6 weeks. Note that the outer region is negative for collagen type II deposition (arrow). D) Hematoxylin and Eosin (HE), Safranin Red (sGAG), and Picrosirius Red (Collagen) stains of fused iMPC and oMPC assembled microtissues at 6 weeks. Note a stronger intensity of sGAG in the inner core of the construct (arrow) and note a stronger intensity of collagen in the outer region of the construct (arrow). E) Biochemical quantification of total DNA, sGAG, collagen, sGAG/DNA, and collagen/DNA of fused iMPC and oMPC assembled microtissues at 6 weeks. N = 3, mean ± SD. The asterisks indicate p‐values obtained by a nonpaired student t‐test (^*^ *p* <0.05). Scale bars: (A) −100 µm; (B) 5 mm; (C and D): 500 µm.

The meniscus is zonally divided into inner and outer regions with phenotypically different compositions,^[^ ^12^ ^]^ and recapitulation of this zonal organization is believed to be integral to the success of meniscus tissue engineering. Therefore, we next sought to biofabricate a construct with regionally distinct matrix phenotypes resembling the native meniscus by spatially localising iMPC and oMPCs derived microtissues within a supporting mould (**Figure** **7**). To this end we initially separately assembled iMPC and oMPC derived microtissues (2000 cells µT^−1^), first allowing the iMPC microtissues to fuse in a cylindrically shaped mould, while the oMPC microtissues were allowed to fuse in a ring‐shaped mould. After 5 days of culture, pre‐fused iMPC and oMPC microtissue‐derived constructs were assembled in an agarose coated well and maintained for a further 6 weeks of culture to generate a cohesive graft (graphical abstract figure D).

Phase contrast images revealed that there was a noticeable separation between the iMPC and oMPC regions after 2 days of fusion (Figure 7A, arrow), however by the end of the 6 weeks culture period, the two zones of the construct were fully integrated (Figure 7A, arrow). Safranin‐O staining suggested greater sGAG deposition in the core of the final assembled construct (Figure 7D, arrow) when compared to the outer region. In addition, staining for collagen deposition was more intense in the outer regions of the construct (Figure 7D, arrow). The assembled graft stained positive for type I in both the inner and outer regions (Figure 7C). In contrast, there was no evidence for type II collagen deposition in the outer regions of the assembled graft and in the internal core (Figure 7C, arrow). No significant difference was observed in the sGAG/DNA content; however, the collagen/DNA ratio was significantly higher (*p* <0.01) in the oMPC region (Figure 7E).

## 3 Discussion

In this study, we report for the first time the biofabrication of zonally defined meniscus grafts through the assembly of phenotypically distinct fibrocartilage microtissues. Microtissues were successfully fabricated using progenitor cells isolated from both the inner (iMPC) and outer (oMPC) regions of the meniscus, with both cell types displaying robust fibrochondrogenesis and distinct meniscus region‐specific phenotypes. Furthermore, we observed that iMPC and oMPC‐derived microtissues could fuse in various spatial configurations, maintaining shape fidelity and generating an extracellular matrix rich in sGAG and type I collagen. We also found that cABC treatment during tissue development modulates collagen fiber formation and organization. Finally, to demonstrate the utility of this biofabrication strategy, we engineered a cohesive yet zonally defined meniscal graft through the assembly of iMPC and oMPC‐derived assembled microtissues. Such grafts may form the basis of new treatments for damaged or diseased meniscal tissue.

The biofabrication of living meniscal grafts relies on the identification of a suitable cell source with robust fibro‐chondrogenic potential. The observation that tissue‐resident stem/progenitor cells play a critical role in organ homeostasis and wound healing have motivated their use in tissue engineering and regenerative medicine. Meniscus resident progenitor cells possess an inherent meniscal phenotype and intrinsic regenerative capabilities, motivating their use in emerging meniscus tissue engineering strategies.^[^ ^36^ ^]^ Similar to previous studies,^[^ ^18^ ^]^ we found that caprine meniscus progenitor cells can be isolated via differential fibronectin adhesion from both the inner (iMPC) and outer (iMPC) regions of the meniscus. These cells exhibit enhanced colony formation potential, robust proliferative capacity, and the ability to differentiate toward osteogenic, adipogenic, and (fibro)chondrogenic lineages, while maintaining a zonal fibrochondrogenic phenotype that outperforms that of non‐selected fibrochondrocytes (iFC and oFC). This aligns with other studies which highlight the superior clonogenicity, proliferative potential, and multipotent differentiation ability of MPCs isolated from vascular and avascular regions compared to the non‐fibronectin adherent meniscus cell population which lack these capabilities.^[^ ^34^, ^35^ ^]^

We then explored the potential of MPCs for the biofabrication of region‐specific microtissues, with the goal of engineering meniscal grafts capable of mimicking the unique zonal nature of the native tissue. The outer meniscus is rich in aligned type I collagen fibers arranged circumferentially to better withstand tensile forces, while the inner zone contains a higher content of sGAGs and type II collagen to resist compressive loading.^[^ ^45^, ^46^ ^]^ Our study demonstrated that both iMPC and oMPC can be used to biofabricate microtissues that recapitulate certain region‐specific characteristics of the native meniscus. We initially explored different cell densities, and as expected, increasing the initial cell numbers (from 1000 to 4000 cells microtissue^−1^) led to the formation of slightly larger microtissues, although these differences were not dramatic. Previous studies have also investigated the influence of cell density on the development of hyaline cartilage microtissues, highlighting how the initial cell density can modulate microtissue growth.^[^ ^30^ ^]^ Other scaffold‐free meniscus tissue engineering strategies have found that lower initial seeding densities support superior mechanical and biochemical properties, with improved collagen and glycosaminoglycan content.^[^ ^47^ ^]^ In agreement with these findings, we observed that matrix synthesis within the larger ring‐shaped moulds was higher using lower initial cell densities per microtissue. This suggests that nutrient constraints within the higher cell density constructs is negatively impacting matrix synthesis. These findings suggest that optimizing cell density is integral to improving the properties of such engineered meniscal grafts. Independent of the initial cell density per microtissue, we observed that iMPC microtissues were richer in type II collagen, while oMPC microtissues contained higher levels of type I collagen. Additionally, higher sGAG synthesis was observed in the iMPC‐derived microtissues. Such zonal variations in collagen expression aligns with previous studies showing that MPCs from inner and outer zones can generate anisotropic fibrocartilaginous tissues with distinct phenotypes when bioprinted within melt electrowritten scaffolds.^[^ ^18^ ^]^

One of the key advantages of using microtissues for tissue engineering applications is their capability to fuse and generate scaled‐up grafts.^[^ ^48^, ^49^, ^50^, ^51^ ^]^ After the characterization of iMPC and oMPC microtissues, we next evaluated their potential as building‐blocks in the bioassembly of larger grafts. When iMPC and oMPC microtissues were assembled within a ring‐shaped agarose mold, they generated a tissue with a circumferential aligned collagen network similar to that found in the native meniscus.^[^ ^52^, ^53^ ^]^ These circumferential collagen fibers resist the tensile forces within the meniscus during knee loading.^[^ ^54^ ^]^ The periphery of these scaled‐up grafts was richer in collagen, which is potentially linked to spatial differences in nutrient availability or biomechanical cues (e.g., matrix tension) that develop within the graft over the culture period. As expected, bone marrow MSC microtissue‐derived constructs contracted in culture^[^ ^55^, ^56^ ^]^ and did not fuse into the desired ring shape. Together, these results highlight the benefit of using MPC‐derived microtissues for engineering meniscal grafts. Additionally, modular assembly strategies such as those described here have high potential for automation, which could help meet the clinical demand for scaled‐up grafts.^[^ ^49^ ^]^

Although microtissue assembly enables the biofabrication of extracellular‐matrix‐rich constructs at a millimeter scale, ensuring cohesive microtissue fusion and their (re)modeling into scaled‐up grafts with biomimetic extracellular matrix composition and organization remains a challenge. As previously discussed, the composition and organization of the ECM of the meniscus is integral to its function.^[^ ^74^ ^]^ iMPC‐derived microtissues fuse and generate a more hyaline‐like cartilage tissue that is richer in type II collagen, which should better enable such a tissue to support compressive loading. In contrast, oMPC‐derived microtissues generate a more type I collagen rich ECM, which in the native tissue primarily supports the hoop stresses generated in the outer meniscus. For engineered grafts to replicate the mechanical behavior of the native meniscus, it is critical to recapitulate not only the biochemical composition of the tissue, but also the architecture of each zone.^[^ ^57^, ^58^ ^]^ In this study, we also demonstrate that the temporal introduction of an exogenous remodeling enzyme, chondroitinase‐ABC (cABC), enhanced the intensity of collagen staining, the diameter of collagen fibrils, and the overall organization of the collagen network within the engineered tissue. Numerous studies have explored the use of enzymatic treatments for meniscus engineering.^[^ ^15^, ^16^, ^43^, ^59^, ^60^ ^]^ In line with our findings, Gonzeles‐Leon and collaborators (2020) observed a decrease in sGAG content in the groups treated with cABC, while MacBarb and collaborators (2013) observed the formation of thicker and more organized collagen fibers. Furthermore, Huey and collaborators (2011) observed an improvement in collagen organization with cABC treatment. Other approaches that could be adopted in the future include the addition of an exogenous collagen crosslinking agents such as lysyl oxidase (LOX‐2), in conjunction with cABC, to improve collagen fibril maturity and tissue functionality.^[^ ^12^ ^]^ Taken together, our data suggest a potential benefit in applying cABC treatment to meniscus grafts engineered using MPC‐derived microtissues. Future studies will explore the influence of cABC treatment on the biomechanical properties of such grafts.

Despite numerous studies aimed at developing constructs for meniscus repair,^[^ ^61^, ^62^, ^63^, ^64^ ^]^ replicating the meniscus' heterogeneous composition, including its zonal anatomy, cell phenotype, and region‐specific composition in a single graft remains challenging.^[^ ^65^ ^]^ Previously, we engineered zonally defined meniscus constructs by inkjeting porcine iMPC and oMPC suspensions into MEW scaffolds, resulting in a graft with native‐like extracellular matrix composition and promising mechanical properties.^[^ ^18^ ^]^ In this study, we report the novel assembly of iMPC and oMPC‐derived microtissues to create an integrated meniscus graft. The final construct displayed heterogeneous extracellular matrix composition, mimicking some aspects of the native meniscus. Previous studies have demonstrated the potential of modular assembly techniques for engineering tissues like cartilage,^[^ ^28^ ^]^ bone,^[^ ^66^ ^]^ and blood vessels,^[^ ^67^ ^]^ highlighting the potential of microtissues as building blocks for hierarchical tissue engineering. Our results further support this approach, demonstrating that the spatial assembly of iMPC and oMPC‐derived microtissues supports the development of a meniscus‐like graft. However, it should be noted that total collagen levels within the resulting constructs do not reach native levels, nor have we fully replicated the collagen fiber alignment observed in skeletally mature menisci, both of which are essential for normal tissue function and joint stability. Addressing these limitations, for example through the addition of further mechanical or biophysical stimulation^[^ ^16^, ^68^, ^69^, ^70^, ^71^ ^]^ and/or the incorporation of guiding scaffolding structures^[^ ^18^, ^72^, ^73^ ^]^ will be important for enhancing graft functionality. Future work should also include a comprehensive biomechanical evaluation to fully establish their potential for clinical translation. Incorporating both tensile and compressive testing will offer critical insights into graft functionality, mechanical robustness, and long‐term structural integrity.

In conclusion, this study demonstrates the successful biofabrication of zonally defined meniscus grafts using MPC derived meniscal microtissues. iMPCs and oMPCs isolated from the inner and outer meniscus were highly proliferative and displayed distinct phenotypes. Both iMPC and oMPC derived microtissues were able to fuse and develop region‐specific meniscus tissue when cast into cylindrical and ring‐shaped moulds. Enzymatic treatment with cABC enhanced collagen fiber network maturation and does not affect the capacity of iMPC and oMPCs to generate region‐specific meniscal microtissues, highlighting its potential for optimizing graft properties. The integration of iMPC and oMPC derived microtissues enabled the biofabrication of a scaled‐up, zonally defined and cohesive meniscus graft. Overall, these findings highlight the translational potential of meniscal microtissues‐derived grafts for meniscus repair applications.

## 4 Experimental Section

### Bone Marrow Mesenchymal Stem/Stromal Cells (MSCs) Isolation and Expansion

MSCs were isolated from the sternum of skeletally mature, female, Saanen goats as described previously (18–19). The caprine cells were isolated from waste tissue taken from animals euthanized as part of a separate, regulatory approved project. Briefly, after the bone marrow pieces were harvested, they were gently cut into small pieces using a 10A scalpel. Next, the marrow pieces obtained were vortexed for 5 min in expansion medium (X‐Pan) made from: Dulbecco's Modified Eagle Medium (DMEM) + 100 U mL^−1^ penicillin + 100 µg mL^−1^ streptomycin (Gibco) + 10% (w/v) Fetal Bovine Serum (FBS) + Basic Fibroblastic Growth Factor 2 (FGF‐2), to aid in liberating the cellular components. Then, the culture medium containing the cell suspension was aspirated and passed through a 40 µm cell strainer prior to counting and plating at a density of 5.7 × 10^4^ cells cm^−2^ and expanded under physioxic conditions (37 °C in a humidified atmosphere with 5% CO_2_ and 5% O_2_) for improved chondrogenic differentiation. When the confluency in the flasks was at 80%, MSCs were trypsinized using 0.25% (w/v) trypsin Ethylenediaminetetraacetic acid (EDTA). For microtissues culture, MSCs were expanded from an initial density of 5000 cells cm^−2^ in X‐Pan medium under physioxic conditions until passage 3.

### Meniscus Cell Isolation and Fibronectin Selection

Medial and lateral menisci were obtained from both the left and right knees of three skeletally mature, female, Saanen goat. The caprine cells were obtained during a tissue isolation procedure from animals euthanized as part of a separate, ethics‐approved project by the Irish Health Products Regulatory Authority (approval number AE18982). Under sterile conditions, the menisci were rinsed twice with Dulbecco's phosphate buffer (PBS) containing 100 U mL^−1^ penicillin and 100 µg mL^−1^ streptomycin (P/S) (Sigma‐Aldrich, Dublin, Ireland). The tissue samples were then incubated in pronase (500 U mL^−1^, 30 min at 37 °C), after which the menisci were dissected longitudinally into two parts: the inner zone and outer zones. To increase sample yield, both medial and lateral menisci were used. Each zone was separately minced into 1–2 mm^3^ pieces and digested with collagenase type I solution (900 U mL^−1^, 5 h at 37 °C) (Gibco). Following digestion, the solution was filtered through a 40 µm porous membrane, centrifuged, and resuspended in XPAN medium, consisting of DMEM GlutaMAX supplemented with 10% v/v FBS, 100 U mL^−1^ penicillin, and 100 µg mL^−1^ of streptomycin. Cells extracted from the inner and outer meniscal zones were cultured under two conditions: The first aimed to isolate a mixed population of inner (iFCs) and outer (oFCs) fibrochondrocytes, expanded at a high cell density (≈6000 cells cm^−2^). The second focused on isolating progenitor cells from the inner (iMPC) and outer (oMPC) meniscal region, expanded at a lower density (≈300 cells cm^−2^). These cells were subjected to a fibronectin‐selective adhesion, as previously described.^[^ ^14^ ^]^ Briefly, petri dishes were coated with 10 µg mL^−1^ fibronectin in PBS containing 1 mm MgCl_2_, and 1 mm CaCl_2,_ and incubated overnight at 4 °C. Cells from each region were seeded and incubated for 20 min at 37 °C. Non‐adherent cells were removed, and a fresh XPAN medium with 5 ng mL^−1^ FGF‐2 (PeproTech, UK) was added. The adherent cells were then cultured at 37 °C and 5% CO_2_. This experiment was performed using 2 different female goat donors.

### Fibrochondrogenic Differentiation

The fibrochondrogenic potential of progenitor groups was assessed at passages 2 and 4 using a 3D pellet culture protocol (Barcelo et al., 2023). Briefly, 2.5 × 10^5^ cells were centrifuged into pellets in chondrogenic media (CDM+), consisting of hgDMEM supplemented with (100 U mL^−1^) penicillin, streptomycin (100 µg mL^−1^)(both Gibco), sodium pyruvate (100 µg mL^−1^), L‐proline (40 µg mL^−1^), L‐ascorbic acid 2‐phosphate (50 µg mL^−1^), linoleic acid (4.7 µg mL^−1^), bovine serum albumin (1.5 mg mL^−1^), Insulin‐Transferrin‐Selenium, dexamethasone (100 nm), Amphotericin B (2.5 µg mL^−1^) (all from Sigma), and human transforming growth factor‐β3 (TGF‐β) (10 ng mL^−1^) (Peprotech). After 21 days in physioxic (37 °C, 5% CO_2_, 5% O_2_), three pellets per cell region were used for biochemical quantification of DNA, sGAG, and collagen, while two were fixed in 4% paraformaldehyde (PFA) for histological analysis.

### Osteogenic and Adipogenic Differentiation

Cells from the meniscus (iMPC, oMPC, iFC, and oFC) were seeded at ≈1 × 10^3^ cells cm^−2^ into 6‐well plates and cultured in XPAN medium for 2–3 days. After reaching an 80% confluency, the basic medium was replaced with osteogenic medium [XPAN + 100 nm dexamethasone, 10 mm *β*‐glycerophosphate, and 0.05 mm ascorbic acid (all from Sigma)] and cultured for 21 days with media refreshed twice per week. For controls, cells were maintained in XPAN for the duration of culture. On day 21, cells were washed twice with PBS, fixed in iced ethanol at RT for 10 min, washed again with PBS and distilled water. Alizarin Red (1%, Sigma) was added for 1 min, followed by a final rinse with distilled water before phase contrast imaging (Zeiss) to investigate calcium deposition.

For adipogenic differentiation, cells were seeded at the same density as previously described for the osteogenic differentiation. The adipogenic medium was supplemented with 100 nm dexamethasone, 0.5 mm IBMX, and 50 µm indomethacin (all from Sigma). After 21 days in culture, cells were washed with PBS, incubated in 0.6% Oil Red O (ORO) solution at RT for 30 min, and washed with PBS until the background was clear. Positive adipogenic differentiation was confirmed by Oil red staining covering over 30% of the monolayer, evaluated by phase contrast microscopy (Zeiss).

### Microtissue Biofabrication and Culture

iMPC, oMPC, and MSC‐derived microtissues were fabricated by using an in‐house high‐throughput non‐adherent agarose hydrogel microwell system as described previously.^[^ ^18^, ^19^ ^]^ In this system, it is possible to fabricate up to 1889 microtissues at once. For microtissue formation using a 400 microwells mould^−1^, cells at passage 3 were seeded on top of each mould at final densities of 1 × 10^3^, 2 × 10^3^, or 4 × 10^3^ cells microwell^−1^. For microtissue formation using an 1889 microwells mould^−1^, a density up to 3 × 10^3^ cells microtissue^−1^ was seeded in X‐Pan media. After 24 h, in order to allow cell aggregation, the microtissues were maintained in chondrogenic induced culture conditions consisting of hgDMEM GlutaMAX supplemented with 100 U mL^−1^ penicillin, 100 µg mL^−1^ streptomycin (both Gibco), 100 µg mL^−1^ sodium pyruvate, 40 µg mL^−1^ L‐proline, 50 µg mL^−1^ L‐ascorbic acid‐2‐phosphate, 4.7 µg mL^−1^ linoleic acid, 1.5 mg mL^−1^ bovine serum albumin, 1 × insulin–transferrin–selenium (ITS), 100 nm dexamethasone (all from Sigma), 2.5 µg mL^−1^ amphotericin B and 10 ng mL^−1^ of human transforming growth factor ‐ beta 3 (TGF‐β) (Peprotech, UK). The microtissues were cultured under physioxic conditions (37 °C in a humidified atmosphere with 5% CO_2_ and 5% O_2_) for up to 3 weeks using the 400 microwells mould^−1^ and for 2 days using the 1889 microwells mould^−1^. This experiment was performed using cells from two different biological replicates, with comparable results obtained for each. The results from one representative replicate are presented in the manuscript.

### Microtissue Diameter and Sphericity Measurements

iMPC and oMPC microtissues were monitored and images were acquired using the 4x objective of an optical inverted microscope (Primo Vert, Zeiss, USA) equipped with a digital camera. Width and length were measured using the ImageJ 1.53e software (Wayne Rasband and Contributors, USA). A diameter ratio of each microtissue was obtained by dividing width by length (sphericity). The analysis was performed using one cell donor and three independent experiments using fifteen spheroids from each group.

### Fusion Assays

The capacity to form fibrocartilage zonally defined assembled microtissues by spontaneous self‐assembly was determined by manually seeding the iMPC, oMPC, and MSC microtissues in custom ultrapure non‐adherent agarose (Sigma) moulds. The moulds were created using sterile 2% w/v agarose cast into a 12‐well plate. The central agarose well of the cylindrical mould was 3 mm in diameter and 1.5 mm in depth, while the central agarose well of the ring mould had an inner diameter of 5 mm, an outer diameter of 8 mm, and a depth of 1.5 mm. The total number of microtissues seeded in the cylindrical‐shape mould was 1700 and, in the ring‐shape mould was 3400. Each well was then topped up with 3 mL of induced medium and cultured in physioxic conditions (37 °C in a humidified atmosphere with 5% CO_2_ and 5% O_2_) for up to 4 weeks.

A modified version of this fusion assay was also developed to allow the assembly of iMPC and oMPC‐derived microtissues in a single graft. To this end, iMPC and oMPC microtissues were fabricated in a density of 2 × 10^3^ cells microwell^−1^. After 48 h, a total of 3400 iMPC microtissues were manually assembled in the cylindrical mould, and the same amount of oMPC microtissues were manually assembled in the ring mould. After 5 days, iMPC and oMPC assembled microtissues were then assembled together in a 2% (w/v) agarose‐coated 6‐well plate, to avoid cell attachment, and a final volume of 6 mL of induced media was added in each well. Constructs were cultured in physioxic conditions (37 °C in a humidified atmosphere with 5% CO_2_ and 5% O_2_) for up to 6 weeks. This experiment was performed using cells from two different biological replicates, with comparable results obtained for each. The results from one representative replicate are presented in the manuscript.

### Chondroitinase‐ABC Treatment

On day 14, prior to enzymatic treatment, iMPC and oMPC‐derived constructs were washed three times in hgDMEM. Following which, they were maintained in an enzymatic solution containing 2 U mL^−1^ cABC (Sigma‐Aldrich) and 0.05 m acetate (Trizma Base, Sigma‐Aldrich) activator in hgDMEM for 4 h in physioxic conditions.^[^ ^44^ ^]^ After the treatment, the samples were washed again three times with hgDMEM to ensure removal of any residual cABC before the addition of fresh induced medium and the continuation of cultivation in physioxic conditions for the remaining 2 weeks. This experiment was performed using cells from two different biological replicates, with comparable results obtained for each. The results from one representative replicate are presented in the manuscript.

### Histological Evaluation

Briefly, samples were fixed using 4% (w/v) paraformaldehyde (PFA) solution (Sigma) overnight at 4 °C. After fixation, samples were dehydrated in a graded series of ethanol solutions (70–100%), cleared in xylene, and embedded in paraffin wax (all Sigma). Tissue sections (5 µm) were taken at the centre of the defect in the transverse plane using a manual microtome (Leica) and rehydrated before staining. Sections were stained with haematoxylin and eosin (HE) for morphology evaluation, 1% (w/v) alcian blue 8GX in 0.1 m hydrochloric acid (HCL) (AB) to visualize sulphated glycosaminoglycan (sGAG) and counter‐stained with 0.1% (w/v) nuclear fast red to determine cellular distribution, 0.1% (w/v) picrosirius red (PSR) for collagen deposition, and 1% (w/v) alizarin red (pH 4.1) to identify calcium deposition (all from Sigma). Stained sections were then imaged using an Aperio ScanScope slide scanner (Leica).

### Immunohistochemistry Analysis

Immunohistochemistry analyses were performed at the end of the culture period to assess differences in the phenotype between the iMPC and oMPC microtissues and derived constructs. The analyses were performed for collagen I (Abcam ab90395 1:400) and collagen II (Santa Cruz sc52658 1:400) as previously described.^[^ ^38^ ^]^

### Polarized Light Microscopy (PLM) and Collagen Alignment Quantification

Sections stained with picrosirius red staining were imaged using PLM to visualize collagen fiber orientation. Quantification of mean fiber orientation, fiber dispersion, fiber coherency, and the generation of color maps was carried out using previously established methods^[^ ^37^, ^39^ ^]^ utilizing the “directionality” feature in ImageJ *software* as well as the OrientationJ plugin.

### Biochemical Analysis

After harvested, samples were washed 2 times in phenol‐free DMEM (pfDMEM, Sigma–Aldrich, Wicklow, Ireland) media and manually dried. Next, 3.88 U mL^−1^ of papain enzyme in 100 mm sodium phosphate buffer/5 mm Na2EDTA/10 mm L‐cysteine, pH 6.5 (all from Sigma–Aldrich), was used to digest the samples at 60 °C for 18 h using a rotator. The completely digested samples were then vortexed for 1 min and centrifuged for 5 min at 650 g.

The DNA content was quantified immediately after digestion using the Quant‐iT PicoGreen dsDNA Reagent and Kit (Molecular Probes, Biosciences) following the company recommendation steps. The DNA content of each sample was quantified using the Synergy HT multi‐detection microplate reader (BioTek Instruments, Inc) at a wavelength of 480 nm.

The amount of glycosaminoglycans (sGAG) was determined using the dimethylmethylene (DMMB) blue dye‐binding assay, with a chondroitin sulphate solution (Blyscan, Biocolor Ltd., Carrickfergus, UK) for the standards. The pH of the DMMB was adjusted to 1.5 using a 12 N HCl solution. The samples were read using the Synergy HT multi‐detection microplate reader (BioTek Instruments, Inc) at a wavelength of 530 and 590 nm.

The total collagen content was obtained using a chloramine‐T assay. Briefly, samples were initially mixed with 38% HCL (Sigma) and incubated at 110 °C for 18 h to allow hydrolysis to occur. Samples were subsequently dried in a fume hood and the sediment reconstituted in ultra‐pure water. A concentration of 2.82% (w/v) Chloramine T and 0.05% (w/v) 4‐(Dimethylamino) benzaldehyde (both Sigma) were then added in the samples and the hydroxyproline content quantified with a *trans*‐4‐Hydroxy‐L‐proline (Fluka analytical) standard using a Synergy HT multi‐detection microplate reader at a wavelength of 570 nm (BioTek Instruments, Inc). The total collagen content was estimated by measuring hydroxyproline levels and applying a hydroxyproline‐to‐collagen ratio of 1:7.69. These experiments were performed using cells from two different biological replicates, with comparable results obtained for each. The results from one representative replicate are presented in the manuscript.

### Scanning Electron Microscopy (SEM)

Briefly, samples were initially fixed in 3% (w/v) glutaraldehyde for 2 h at room temperature and washed twice with 1x PBS. Next, chondroitin sulfate and dermatan sulfate were removed by incubating samples overnight in 0.6 U mL^−1^ cABC prepared in a buffer of 0.05 m tris HCl and 0.06 m sodium acetate at pH 8.0. Hyaluronic acid was then removed by incubating the samples overnight in 20.4 U mL^−1^ hyaluronidase (all from Sigma). Samples were next dehydrated in crescent concentrations of ethanol: 30%, 50%, 70%, 90% and 100% of 10 min each and dried using critical point. The dried samples were mounted on SEM pin stubs with carbon adhesive discs and coated with gold/palladium for 60 s at a current of 40 mA using a Cressington 208 h sputter coater. Imaging was carried out in a Zeiss ULTRA plus SEM. The diameter of the collagen fibers was measured using ImageJ software.

### Statistical Analysis

Statistical analysis was performed using GraphPad Prism software (GraphPad Software, CA, USA). A *two‐way* ANOVA was performed followed by Tukey's multiple comparison post‐test to assess the differences in diameter and sphericity of the microtissues with time in culture and the biochemical content of the tissues. A non‐paired student *t*‐test was performed to compare the differences in biochemical content of iMPC and oMPC grafts. Numerical and graphical results are presented as mean ± standard deviation. Significance was determined when *p <*0.05.

## Conflict of Interest

The authors declare no conflict of interest.

## Supporting information



Supporting Information

## Data Availability

The data that support the findings of this study are openly available in BioRxiv at https://doi.org/10.1101/2025.04.25.650568, reference number 650568.
